# How Do Host–Parasitoid Interactions Shape the Effectiveness of *Anagyrus aberiae* as a Biological Control Agent?

**DOI:** 10.3390/insects17080790

**Published:** 2026-07-29

**Authors:** Elena Romero, Antonia Soto

**Affiliations:** Instituto Agroforestal Mediterráneo (IAM), Universitat Politècnica de València, Camino de Vera s/n, 46022 Valencia, Spain

**Keywords:** host–parasitoid interactions, host-stage selection, population dynamics, functional response, parasitoid, biological control, *Delottococcus aberiae*, *Anagyrus aberiae*

## Abstract

The invasive mealybug *Delottococcus aberiae* (De Lotto) has become a major citrus pest in eastern Spain during the last decade. The parasitoid *Anagyrus aberiae* (Guerrieri) is currently used in a classical biological control program against this pest, although important aspects of the interaction between both species remain poorly understood. We evaluated host-stage suitability and the effects of parasitism on host reproduction, as well as demographic performance and parasitoid response to host density under laboratory conditions. The parasitoid successfully parasitized *D. aberiae* stages from second-instar nymphs to adults, but preferred adult females, which accounted for 86% of parasitism when different stages were offered simultaneously. Parasitism reduced reproductive output by more than 93% in pre-reproductive females and caused oviposition cease within two days in females that had already initiated egg laying. *Anagyrus aberiae* exhibited an intrinsic rate of increase (r_m_) of 0.099, and a Type II functional response, reaching a maximum parasitism rate of 8.7 hosts per day. The strong suppression of host reproduction and favorable population growth parameters highlight the biological control potential of *A. aberiae*. These findings provide valuable information for optimizing mass-rearing protocols and release strategies, thereby improving the implementation of biological control programs against *D. aberiae*.

## Simple Summary

*Delottococcus aberiae* is an invasive mealybug that has become an important pest of citrus crops in eastern Spain, causing damage that reduces fruit quality and economic value. To help control this pest, a parasitic wasp, *Anagyrus aberiae*, was introduced from the mealybug’s area of origin in South Africa in 2019. However, little was known about how this natural enemy attacks the pest and how effective it may be under different conditions. In this study, we examined which life stages of the mealybug are most suitable for the parasitoid, how parasitism affects mealybug reproduction, and the ability of the parasitoid population to grow and respond to different pest densities. The parasitoid was able to attack all tested stages of *D. aberiae* but strongly preferred adult females. Parasitized female mealybugs produced fewer eggs than unparasitized control ones, and females already laying eggs stopped doing so within two days after parasitism. The parasitoid also showed a strong capacity for population growth and parasitized more mealybugs as host density increased, although this response slowed at high densities. These findings provide valuable information for improving biological control strategies against *D. aberiae* and for guiding future research on this host–parasitoid system.

## 1. Introduction

Following the detection of *Delottococcus aberiae* (De Lotto) (Hemiptera: Pseudococcidae) in eastern Spain in 2009, this invasive mealybug has become a major citrus pest due to the severe fruit damage it causes, prompting extensive research into its biology and management [1,2]. The problem was exacerbated by the limitations of available management measures. The cryptic habits of *D. aberiae*, which shelters in protected sites, and restrictions on insecticide applications during flowering limit the effectiveness of chemical control, while native natural enemies fail to suppress its populations [3,4]. Consequently, a classical biological control program was initiated using *Anagyrus aberiae* (Guerrieri) (Hymenoptera: Encyrtidae), a newly described parasitoid imported from the native range of the mealybug in South Africa [5,6].

Since then, several field studies have characterized the seasonal abundance, spatial distribution, and damage patterns of *D. aberiae* in citrus orchards [2,4,7]. More recently, laboratory studies examining the thermal biology, reproductive traits, and developmental parameters of both the mealybug and the parasitoid have improved our understanding of their population dynamics and the potential of *A. aberiae* as a biological control agent [8]. However, important ecological aspects underlying the interaction between both species, particularly the consequences of parasitism for host survival and reproduction, remain poorly understood.

The effectiveness of parasitoids as biological control agents is shaped by multiple interactions, including the selection of the host stage for parasitism and parasitic behavior itself, which depends on the density of the host pest [9,10]. Furthermore, from a population perspective, their growth and overall impact on the host population dynamics are also important factors.

Host-stage suitability significantly influences parasitoid development, offspring fitness and sex allocation [11], as host developmental stages differ in the resources they provide and thus in their profitability to parasitoids [12]. In arrhenotokous parasitoids, like *A. aberiae*, fertilized diploid eggs (female offspring) are typically allocated to larger, higher-quality hosts, whereas unfertilized eggs (male offspring) are more often laid in smaller ones [13,14]. In addition, host-stage selection is also influenced by parasitoid developmental strategy. Koinobiont parasitoids, such as *A. aberiae*, usually exhibit a wider range of suitable developmental stages for parasitism, as their hosts continue feeding and developing after being parasitized [12,15]. Consequently, although multiple host stages may be suitable for parasitoid development, they may differ in their value for offspring production and fitness. Despite the extensive literature on host-stage preference in parasitoids, comparatively less attention has been given to the reproductive responses of hosts following parasitism, particularly when parasitism occurs at advanced developmental stages [15,16]. This aspect is particularly relevant when parasitism occurs during late developmental or reproductive stages of the host. In such cases, female hosts may continue egg maturation and oviposition after being parasitized, potentially reducing the impact of parasitism on pest suppression [15,17]. Therefore, assessing the effect of parasitism on late developmental stages of *D. aberiae* would allow determination of whether *A. aberiae* can completely suppress host reproduction or, alternatively, the extent of the reduction in reproductive output, thereby helping to evaluate its potential as a biological control agent.

Another factor influencing the effectiveness of a parasitoid is its ability to develop and reproduce on its hosts. Demographic traits such as survival, fecundity, and intrinsic rate of increase determine the capacity of a parasitoid to sustain population growth and maintain pest suppression over time [11,18]. Moreover, demographic analyses allow comparisons between the population growth potential of parasitoids and their hosts, providing insights into the ability of natural enemies to respond to increases in pest populations [19]. While host-stage suitability and demographic traits determine how effectively parasitoids exploit and develop on hosts, varying host densities influence their foraging behavior and parasitism efficiency. Functional response, which describes the parasitic behavior of an individual parasitoid to varying host density, is a very widely used model to evaluate parasitoid performance and predict host suppression capacity [12,20,21,22]. Three types of functional response have been described [21]. Type I functional response describes a linear relationship between the number of parasitized hosts and host density. Type II response is characterized by a hyperbolic curve in which the proportion of parasitized hosts decreases with increasing host density, whereas Type III response follows a sigmoidal pattern where the proportion of parasitized hosts initially increases before reaching saturation at higher host densities. Type II and Type III are the most commonly reported in parasitoids and differ in their implications for host–parasitoid dynamics [23,24,25]. Therefore, determining the type of functional response of the parasitoid is important for evaluating its regulatory effect on host populations [26,27,28].

This study represents the first comprehensive evaluation of the host exploitation strategy of *A. aberiae*, combining host-stage suitability, host reproductive suppression, functional response, and demographic performance. Together, these findings improve our understanding of the biological mechanisms determining the effectiveness of *A. aberiae* against *D. aberiae* in citrus of eastern Spain. They will also contribute to optimizing mass-rearing protocols by identifying the host stages and rearing conditions that maximize parasitoid production while improving release strategies.

## 2. Materials and Methods

### 2.1. Insect Colonies

A laboratory colony of *D. aberiae* was established in an insectary at the Universitat Politècnica de València (UPV) using field-collected material from citrus orchards in Les Valls (Valencia, Spain). Mealybugs were reared on pumpkins, *Cucurbita maxima,* previously washed in a 2% bleach solution to prevent fungal growth. Infested pumpkins were placed in sleeve cages (47.5 × 47.5 × 47.5 cm), each containing 3 to 5 pumpkins. To maintain the colony, 3 to 4 fresh pumpkins were introduced every two weeks to replace the dried ones. These were infested by manually transferring egg sacs from the old pumpkins using a fine camel hair brush. The colony was kept in darkness in a climate-controlled chamber (MLR-351; SANYO Electric Co., Ltd., Moriguchi, Osaka, Japan) at a temperature of 24 °C and 70% ± 10 RH.

Rearing of *A. aberiae* was initiated from parasitoid individuals introduced from South Africa in 2020 and maintained on *D. aberiae*-infested pumpkins inside sleeve cages as described above. Infested pumpkins were renewed every 3 weeks to ensure a continuous supply of mealybugs. Parasitoids were fed drops of diluted honey through the cage walls three times a week. The colony was kept in a climate-controlled chamber at 24 °C, 70% ± 10 RH and a photoperiod of 14:10 L:D.

For experiments, bean pods were inoculated with 40 mature female mealybugs collected from the colony and placed in 1.6 L plastic containers, and 40 pairs of newly emerged *A. aberiae* were introduced for 24 h. The parasitoids were then removed, and the containers incubated. Mummified mealybugs were checked daily, and newly emerged parasitoids were collected, sexed, and kept individually in glass vials with a drop of honey and a cotton plug until used. The number of containers was adjusted to obtain the required number of parasitoids for the experiments. A target of 20 replicates was established for each experiment. Replicates lost due to accidental mortality or other technical issues were excluded, while maintaining sufficient sample size for statistical analyses.

### 2.2. Parasitism Response of A. aberiae to Different D. aberiae Developmental Stages

The suitability and preference of different *D. aberiae* developmental stages for *A. aberiae* parasitism were evaluated using no-choice tests (single-host stage) and choice tests (mixed-host stages). Three stages were considered, second-instar nymphs (approximately 15 days after egg hatching), third-instar nymphs (26 days), and adult females, which were subdivided into young (virgin; 38 days old) and mature (mated; 54 days) females, based on the developmental times reported by Romero et al. [29]. Experiments were conducted in arenas consisting of a bean pod placed inside a 90-mm Petri dish.

In the no-choice tests, 16 individuals of a single *D. aberiae* stage were transferred onto the bean pod, whereas in the choice tests, 16 individuals were used, comprising four individuals from each stage. Mealybugs were allowed to settle for 1 h before the introduction of one newly emerged (<48 h), mated *A. aberiae* female. The parasitoid was allowed to forage and parasitize for 24 h, after which it was removed and the dishes incubated until mealybug mummification and subsequent parasitoid emergence. The number of parasitized hosts and the offspring sex ratio (calculated as the number of female offspring divided by the total number of offspring) were recorded for each host stage in both experimental types. A total of 18 replicates per test type were conducted under controlled conditions of 24 °C, 70% ± 10 RH and 14:10 L:D.

### 2.3. Effect of Parasitism at Different Adult Female Phases on D. aberiae Survival and Reproduction

**Figure 1:**
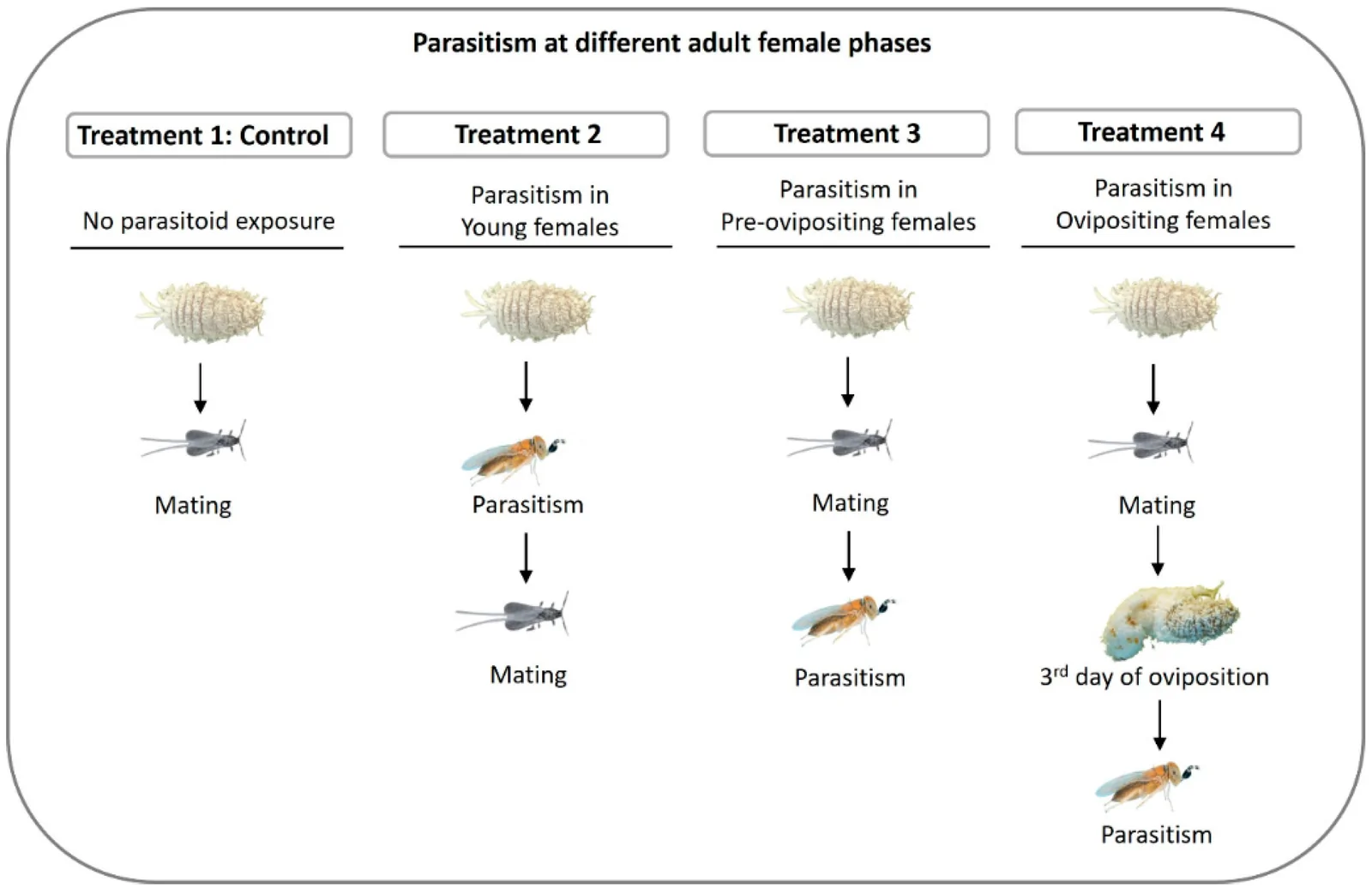
Experimental treatments depending on the parasitization time of *D. aberiae* adult females.

The effect of *A. aberiae* parasitism on host survival and reproduction was evaluated at different physiological phases of adult female *D. aberiae*, representing distinct mating and reproductive conditions. For this purpose, both young (virgin) and mature (mated) adult females were used, with mature females further subdivided into pre-ovipositing and ovipositing females. Four adult female statuses were considered for the experiment (Figure 1): (1) control (unparasitized females); (2) young females (parasitized before mating and subsequently allowed to mate; approximately 4 days after the molt to adulthood); (3) pre-ovipositing females (mature females parasitized before the onset of oviposition; approximately 8 days after the molt to adulthood); and (4) ovipositing females (mature females parasitized on the third day after the start of oviposition; approximately 19 days after the molt to adulthood). Females assigned to each treatment were individually transferred to 1 L plastic containers containing a green lemon fruit, allowing the entire oviposition period to be monitored. In all treatments, three newly emerged mealybug males were provided to ensure mating. Exposure to males in all treatments and to parasitoids in the parasitized treatments lasted 24 h, after which they were removed. Containers were maintained throughout the entire oviposition period, and egg production was recorded daily. Although time-consuming, this approach was feasible. Experiments were replicated at least 12 times per treatment and conducted under controlled conditions of 24 °C, 70% ± 10 RH, and 14:10 L:D.

### 2.4. Population Growth Potential of A. aberiae on Mature Adult D. aberiae Females

To perform a life table analysis and estimate the population growth parameters of *A. aberiae* on adult female *D. aberiae*, a newly emerged female parasitoid was paired with a single male and introduced into a 60-mm Petri dish containing a bean with 5 mature female mealybugs. The parasitoids were provided with a new Petri dish with 5 new mealybugs every day until the female parasitoid died. The parasitized mealybugs were incubated until parasitoid emergence, of which the number and sex of the offspring were recorded. If a male parasitoid died before the female, it was replaced with a newly emerged one. Survival and fecundity curves were constructed using the observed age-specific survival (*l_x_*) and fecundity (*m_x_*) rates, where *x* represents female age (days), *l_x_* the proportion of females surviving to age *x*, and m_x_ the mean number of female offspring produced per female that age. These data were then used to estimate the following demographic parameters [30]: net reproductive rate (*R*_0_ = ∑(*l_x_·m_x_*)), which reflects population increase per generation; intrinsic rate of increase, obtained from the Euler–Lotka equation (*r_m_* = ∑e^−*rm·x*^*·l_x_·m_x_* = 1) and representing the potential population growth; mean generation time (*T* = ∑(*x·l_x_·m_x_*)/*R*_0_), defined as the average female age at reproduction; finite rate of increase (*λ* = e*^rm^* ), corresponding to the daily population multiplication factor; and the doubling time (*DT* = ln(2)/*r_m_*) which represents the time required by a population to double in size. A total of 17 replicate female parasitoids were recorded at 24 °C, 70% ± 10 RH and a photoperiod of 14:10 L:D.

### 2.5. Parasitism Response of A. aberiae to Different D. aberiae Densities

#### 2.5.1. Experimental Design

The effect of host density on *A. aberiae* parasitism was evaluated at 7 densities: 2, 5, 10, 15, 20, 40, and 60 mature *D. aberiae* females. For each host density, the specific number of mealybugs was transferred onto a bean pod and placed in a 90-mm Petri dish, where they were allowed to settle for 1 h before the experiment started. One newly emerged (<48 h) mated female parasitoid was introduced into the arena and allowed to parasitize hosts for 24 h, after which it was removed, and the dishes were incubated upon parasitoid emergence. The number of parasitized mealybugs and the sex ratio of the offspring were recorded at each host density. At least 20 replicates were conducted for each density under controlled conditions of 24 °C, 70% ± 10 RH, and 14:10 L:D.

#### 2.5.2. Analysis of the Functional Response

To determine the type of functional response of *A. aberiae* at different host densities, the analysis was conducted in two steps. First, a logistic regression analysis of the proportion of parasitized hosts as a function of initial host density was used to determine the type of functional response by fitting the polynomial function as described by [31]:

NaN0= exp P0+P1N0+P2N02+P3N031+exp (P0+P1N0+P2N02+P3N03)

where *N*_0_ is the initial number of hosts offered; *N_a_* is the number of parasitized hosts; and *P*_0_, *P*_1_, *P*_2_ and *P*_3_ are the intercept, linear, quadratic and cubic parameters of the curve slope, respectively. These parameters were calculated using the maximum likelihood method. The signs of *P*_1_ and *P*_2_ were used to determine the shape of the curve, and thus the type of functional response. Type I responses are described by an intercept (*P*_0_ > 0) and a constant positive slope (*P*_1_ > 0). When the linear coefficient is significantly negative (*P*_1_ < 0), the parasitoid displays a type II functional response, whereas a type III is indicated by a significantly positive linear coefficient (*P*_1_ > 0) and a negative quadratic value (*P*_2_ < 0).

In the second step, and since the data was classified as a type II (see Section 3), a nonlinear least squares regression was used to estimate the functional response parameters (*T_h_* and *a*) using Rogers’ random parasitoid equation, which is the most appropriate type II functional response equation in cases without host replacement [32,33]:

$$N_{a}=N_{0}\{1-\exp\left[a\left(T_{h}N_{a}-T\right)\right]\}$$

where *T* is the total time that the parasitoid and host were exposed to each other (in this experiment, 24 h), *a* is the attack rate, and *T_h_* is the handling time (h). The maximum number of hosts that can be parasitized by an individual in 24 h (*T*/*T_h_*) was calculated using the estimated *T_h_*.

Regression analyses were used to compare the number of parasitized hosts recorded at different densities. Since the data could not be adjusted to a normal distribution, a Kruskal–Wallis test followed by Dunn’s post-hoc test with Bonferroni’s correction (*p* < 0.05) was used.

### 2.6. Statistics

In the choice and no-choice experiments, the effect of host stage on the number of parasitized and emerged mealybugs, and the sex ratio of offspring were compared using an analysis of variance (ANOVA) with treatment means separated with Tukey’s HSD test at a significance threshold of 0.05. Prior to ANOVA, normality of the residuals and homogeneity of variances were assessed using the Shapiro–Wilk and Levene’s tests, respectively. If necessary, variables were transformed using an arcsine transformation before analysis to satisfy these assumptions. When data could not be adjusted to a normal distribution, a Kruskal–Wallis test was used, followed by Dunn’s post-hoc test using Bonferroni’s correction (*p* < 0.05). Likewise, female longevity and the number of eggs laid in the experiment of parasitism during adult female stages of *D. aberiae* were compared using a Kruskal–Wallis test followed by Dunn’s post-hoc test using Bonferroni’s correction (*p* < 0.05). The cumulative egg production from day 3 until the end of the oviposition period was compared between parasitized ovipositing females and control females using a Student’s *t*-test (*p* < 0.05). Demographic parameters (*R*_0_, *r*_m_, *T*, *DT*, and *λ*) were calculated using the life table data, and standard errors were estimated using the jackknife procedure [34]. All statistical analyses were performed in R version 4.3.2 and RStudio version 2023.12.1 (Build 402; Posit Software, Boston, MA, USA).

## 3. Results

### 3.1. Parasitism Response of A. aberiae to Different D. aberiae Developmental Stages

#### 3.1.1. Host-Stage Suitability and Preference of *A. aberiae*

**Figure 2:**
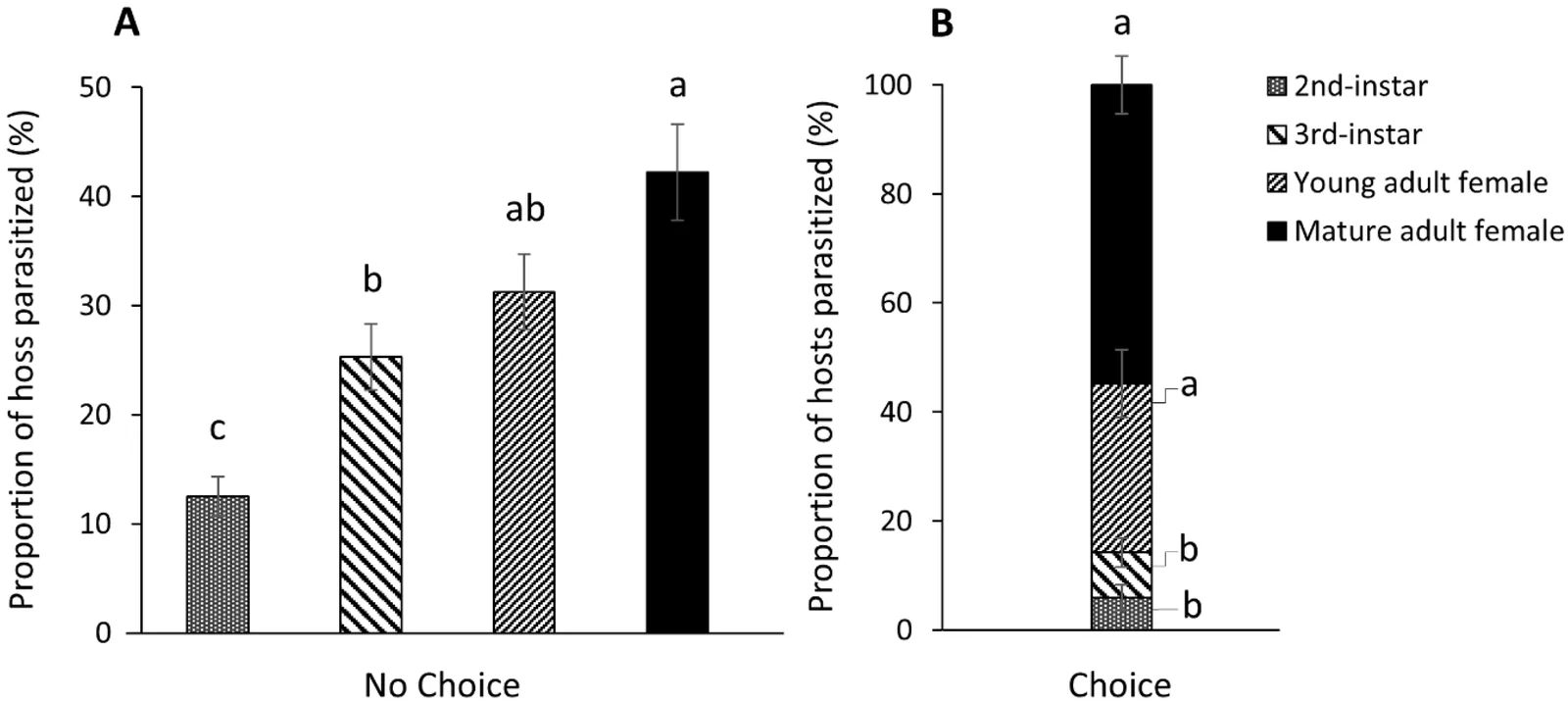
Parasitism of *D. aberiae* in no-choice (**A**) and choice (**B**) tests after a 24 h exposure period. In the no-choice test, each Petri dish contained 16 mealybugs of a single developmental stage to evaluate host-stage suitability for parasitism, and bars represent the mean percentage of hosts parasitized in each host stage. In the choice test, each Petri dish contained 16 mealybugs equally distributed among the four developmental stages (4 individuals per stage) to evaluate parasitoid host-stage preference, and the stacked bar represents the proportion of parasitized hosts corresponding to each developmental stage. Bars topped with different letters indicate significant differences among host stages within the same experimental group (Tukey’s HSD test, *p* < 0.05; Dunn’s test, *p* < 0.05).

*A. aberiae* was able to parasitize all *D. aberiae* developmental stages offered. In the no-choice tests, parasitism increased with host developmental stage, with significantly more adult females being parasitized than second-instar nymphs (*F*_3,79_ = 14.2, *p* < 0.001) (Figure 2A). In the choice tests, adult females were the most preferred hosts, as evidenced by significantly higher parasitism rates than for the second and third-instar nymphal stages (*H* = 42.1, df = 3, *p* < 0.001). On average, young and mature adult females accounted for 31% and 55% of all parasitized hosts, respectively, representing 86% of total parasitism (Figure 2B).

Emergence rates exceeded 80% in all developmental stages and experimental types (Table 1). In no-choice tests, significantly more parasitoids emerged from the third-instar nymphs and adult females (both young and mature) than from second-instar nymphs, with emergence rates exceeding 98% in all three host categories (*H* = 35.6, df = 3, *p* < 0.001). Similarly, in choice tests, adult females achieved significantly higher emergence rates (>96%) than the immature stages (*H* = 39.8, df = 3, *p* < 0.001).

Sex ratio was also significantly influenced by host stage in both tests (no-choice: *H* = 17.6, df = 3, *p* < 0.001; choice: *H* = 27.6, df = 3, *p* < 0.001) (Table 1). In no-choice tests, the sex ratio was female-biased only among mature adult females (60.9% female offspring), whereas among the second instars, it was mainly male-biased (9.4% female offspring). A similar pattern was observed in choice tests, where females emerged mostly from adult female hosts (68 and 69% female offspring), whereas males were predominant in smaller hosts (second-instar nymphs: 25% female offspring) (Table 1).

**Table 1:** Parasitism rate, emergence rate and sex ratio of parasitoids emerging from four *D. aberiae* developmental stages under no-choice and choice tests.

		Immature Stages		Adult Female Stage		*H*	*p*
		2nd-Instar	3rd-Instar	Young	Mature		
No choice	Emergence rate (%)	84.2 b	98.9 a	100 a	98.5 a	33.3	<0.001
	Sex ratio (% ♀)	9.4 a	43.5 ab	44.8 b	60.9 b	17.6	<0.001
Choice	Emergence rate (%)	80 c	85.7 bc	96.2 ab	97.8 a	37.8	<0.001
	Sex ratio (% ♀)	25 a	50 a	68 ab	69 b	27.6	<0.001

Means followed by different letters in a row indicate significant differences among stages within the same experimental group (Dunn’s test, *p* < 0.05). The number of mealybugs available for parasitism was held constant at 16 individuals per patch in both experimental types. ♀ indicates female parasitoids.

#### 3.1.2. Effect of Parasitism at Different Adult Female Phases on *D. aberiae* Survival and Reproduction

Parasitism had a significant effect on oviposition by *D. aberiae* adult females (*H* = 52.0565, df = 3, *p* < 0.001), reducing egg production and, in some cases, completely preventing oviposition (Table 2). While unparasitized adult females (control) were able to lay 253.4 mean eggs throughout their lives, egg production was reduced on all parasitized adult females, regardless of the moment of parasitism (Table 2). This difference was more significant when parasitism was carried out on young and pre-ovipositing mature females, which reduced their oviposition in more than 93% of eggs (0.9 and 16.9 eggs, respectively) compared to the control, and the proportion of females able to lay eggs declined from 100% in the control to 20 and 60% in young and pre-ovipositing mature females, respectively (Table 2).

**Figure 3:**
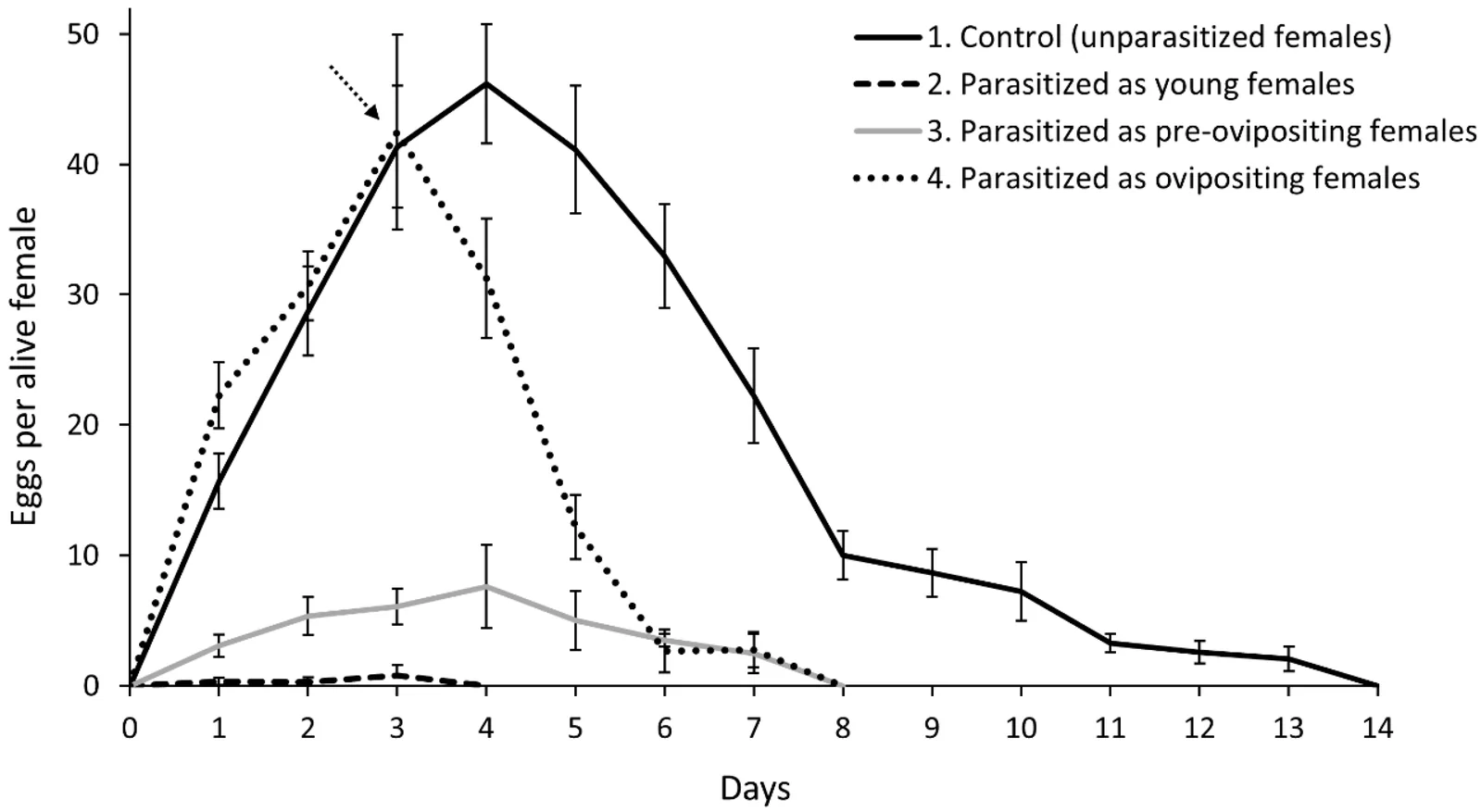
Mean daily egg production of adult female *D. aberiae* parasitized at different physiological stages: (1) unparasitized adult females; (2) young adult females parasitized before mating; (3) mature pre-ovipositing adult females parasitized after mating and before oviposition; and (4) mature ovipositing adult females parasitized on the third day of oviposition (for this treatment, the day of parasitism is indicated by a dashed arrow). Day 0 represents the day preceding the onset of oviposition. Egg laying began on day 1 in all treatments. Error bars represent the standard error of the mean.

In females parasitized on the third day of oviposition (ovipositing mature females), total egg production was 43.7% lower (142.4 eggs; Table 2) than in control females. During the first three days of oviposition, before parasitism occurred, egg production was similar to that of control females (Figure 3). However, when egg production after parasitism was considered (from day 3 of oviposition onwards), parasitized ovipositing females laid significantly fewer eggs than control females during the same period (89.5 vs. 208.9 eggs per female, respectively; Student’s *t*-test: *t* = 4.42, df = 30, *p* < 0.001; Table 2). This result is consistent with the rapid decline in oviposition observed following parasitism (Figure 3).

**Table 2:** Egg production and oviposition performance of *D. aberiae* females parasitized at different reproductive phases.

Adult Female Status at Parasitization	Total Eggs Per Female ± SE	Egg Production Reduction (% vs. Control)	Females Laying Eggs (%)	Mean Ovipositional Period ± SE (Days)	Eggs Per Female After Day 3 ± SE *
1. Control (*n* = 20)	253 ± 18.9 a	0.0	100	10.5 ± 0.4 a	208.9 ± 19.6 a
2. Young (*n* = 15)	0.9 ± 0.8 b	99.6	20	2.3 ± 0.7 b	-
3. Mature pre-ovipositing (*n* = 15)	16.9 ± 5.1 b	93.3	60	4.8 ± 0.5 b	-
4. Mature ovipositing (*n* = 12)	142.4 ± 14.7 a	43.7	100	5.9 ± 0.2 b	89.5 ± 11.9 b
*H*	52.0565			35.246	
*t*					4.4158
*p*	<0.001			<0.001	<0.001

* Calculated only for control and ovipositing females. For ovipositing adult females, day 3 corresponds to the day of parasitoid exposure; for control, the equivalent period was used. Means within a column followed by different letters indicate significant differences among treatments (Dunn’s test, *p* < 0.05; Student’s *t*-test: *t* = 4.41, df = 30, *p* < 0.001).

When parasitism occurred in young adult females or pre-ovipositing mature adult females, oviposition rates remained below 10 eggs per day, and the mean oviposition period ended before day 6, which were significantly shorter than the control treatment (*H* = 35.246, df = 3, *p* < 0.001) (Table 2; Figure 3). When parasitism occurred after oviposition onset, ovipositing females initially showed an oviposition pattern similar to that of the control, reaching a peak of more than 40 eggs on day 3, when parasitism occurred. Thereafter, oviposition declined sharply following parasitism and ceased by day 8 (Figure 3).

### 3.2. Population Growth Potential of A. aberiae Parasitizing Mature Adult D. aberiae Females

**Figure 4:**
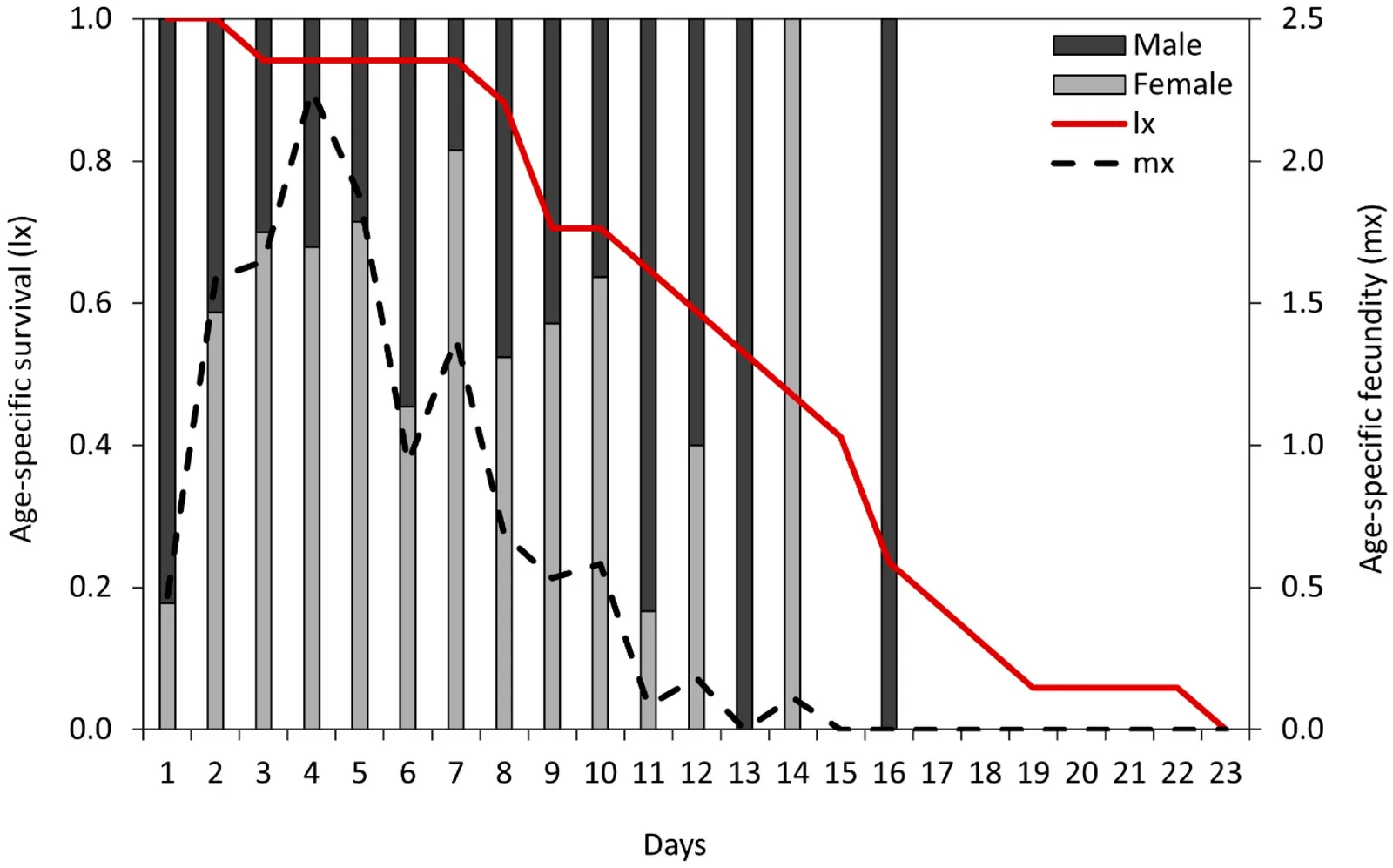
Age-specific survival rate (lx) (y-left axis) and age-specific fecundity (mx) (y-right axis) curves of *A. aberiae*. The age-specific offspring sex ratio is represented in bars (y-left axis).

Survival of *A. aberiae* females (*l*_x_) developed on mature adult *D. aberiae* females steadily declined from the emergence day, with an average female lifespan of 13.41 days (Figure 4). During the first seven days since emergence, 90% of female parasitoids were alive, coinciding with the peak of fecundity (*m*_x_) (Figure 4). *Anagyrus aberiae* oviposition increased rapidly from the emergence day, with the highest fecundity observed between days 2 and 6 and then decreased until it stopped laying eggs on day 15. The sex ratio during the peak of fecundity was predominantly female-biased, with an average of 67% female offspring (Figure 4). From day 7 onwards, both the *l*_x_ and *m*_x_ started to decrease.

The demographic parameters calculated for *A. aberiae* when reared on adult female *D. aberiae* showed a mean lifetime fecundity of 20.41 ± 1.9 eggs per female and a net reproductive rate (*R*_o_) of 11.53 ± 1.7 female offspring per female (Table 3). The intrinsic rate of increase (*r*_m_) was 0.099 ± 0.0006 female offspring per female and day, with a mean generation time (*T*) of 24.622 ± 0.246 days (Table 3).

**Table 3:** Life history and demographic parameters of female *A. aberiae*.

Parameter	Mean ± SE
Total fecundity (total offspring/♀)	20.41 ± 1.9
*R*_o_ (♀/♀)	11.529 ± 1.72
*r*_m_ (♀/♀/day)	0.099 ± 0.006
*T* (days)	24.622 ± 0.246
*DT* (days)	6.981 ± 0.422
*λ* (♀/♀/day)	1.104 ± 0.007

*R*_o_, net reproductive rate; *r*_m_, intrinsic rate of increase; *T*, mean generation time; *DT*, doubling time; *λ*, finite rate of increase. ♀ indicates female parasitoids.

### 3.3. Parasitism Response of A. aberiae to Different D. aberiae Densities

**Figure 5:**
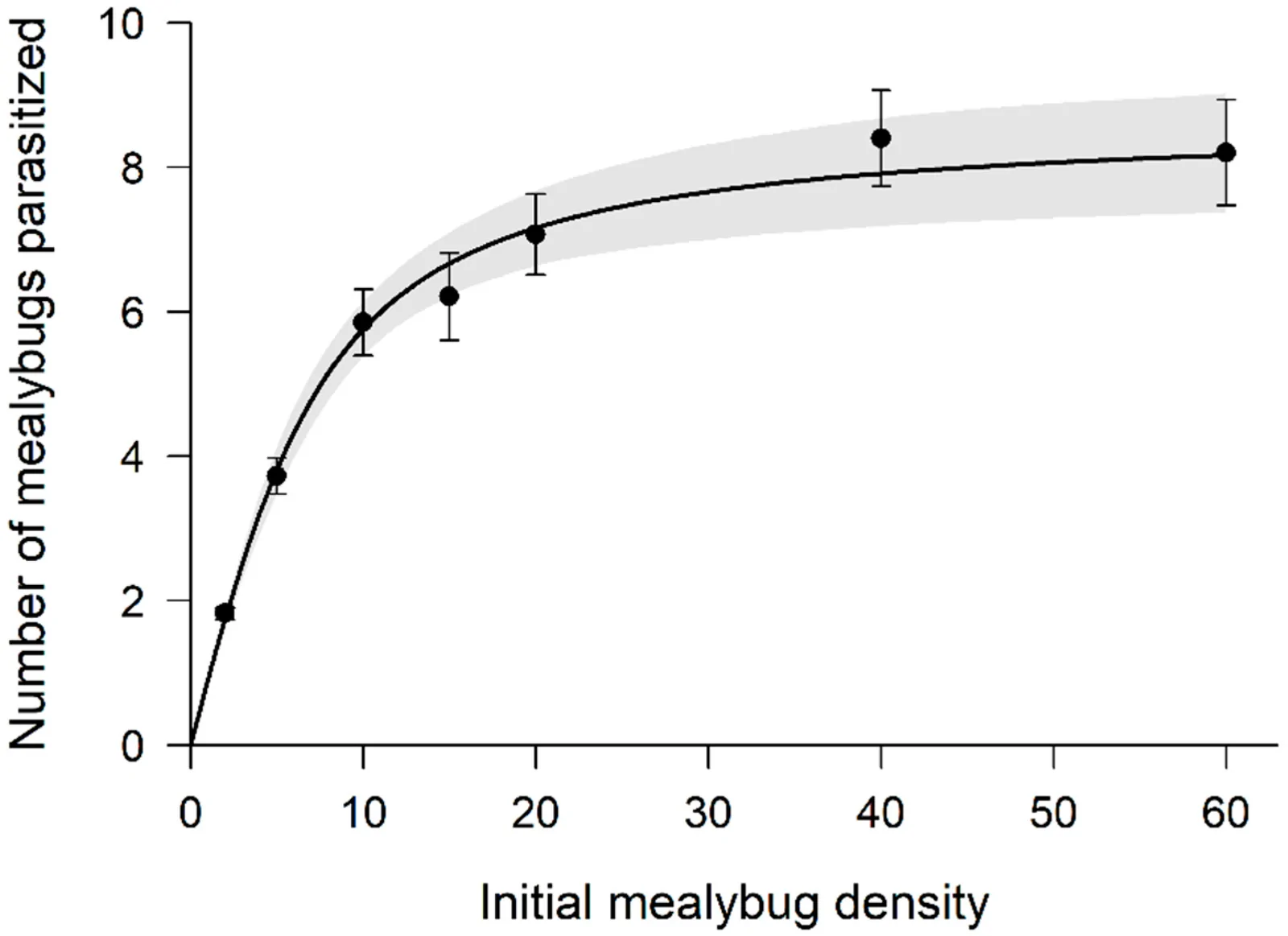
Mean ± SE number of *D. aberiae* parasitized by *A. aberiae* at different host densities within a 24 h period. Shaded areas represent 95% confidence intervals.

*Anagyrus aberiae* exhibited a Type II functional response, as indicated by the logistic regression analysis, which showed a significantly negative linear term coefficient (*P*_1_) (Table 4). The number of mealybug hosts parasitized increased positively with host density until reaching a saturation point, after which the slope of the curve declined at densities above 10 individuals (Figure 5). The overall effect of host density on parasitism rates was significant (*H* = 73.9, df = 6, *p* < 0.001), and the maximum parasitism rates were observed at the greatest densities tested (40 and 60 hosts provided), with 8.4 and 8.2 parasitized hosts, respectively (Figure 5).

The functional response parameters estimated using Rogers’ random parasitoid equation were an attack rate (*a*) of 0.1067 ± 0.0137 h^−1^ and a handling time (*T*h) of 2.7726 ± 0.1299 h (Table 5). Based on these parameters over a 24 h experimental period (*T*), the theoretical maximum number of hosts parasitized per parasitoid female (*T*/*T*h) was estimated at 8.7 hosts per day (Table 5).

**Table 4:** Maximum likelihood estimates from logistic regression analysis on the proportion of *D. aberiae* hosts parasitized by *A. aberiae* females in relation to the initial host density.

Parameter	Estimate	SE	*χ* ^2^	*p*
Constant (*P*_0_)	2.22	0.274	77.9	<0.0001
Linear (*P*_1_)	−0.238	0.0327	373	<0.0001
Quadratic (*P*_2_)	0.00555	0.00108	342	<0.0001
Cubic (*P*_3_)	−0.0000452	0.0000103	334	<0.0001

**Table 5:** Estimate of parameters from Roger’s type II random predation equation.

Parameter	Estimate	Approximate SE	Approximate 95% CI		*T*/*T*h
			Lower	Upper	
*a* (h^−1^)	0.1067	0.0137	0.0798	0.1335	8.7
*T*h (h)	2.7726	0.1299	2.5180	3.0272	

## 4. Discussion

### 4.1. Host-Stage Suitability and Host Reproductive Suppression by A. aberiae

*Anagyrus aberiae* can parasitize a wide range of *D. aberiae* developmental stages but shows a clear preference for adult female mealybugs, which results in higher parasitism rates, emergence rates, and female offspring production at this stage. This pattern is common among parasitoids and has been reported in several *Anagyrus* species, including *Anagyrus kamali* (Moursi), *Anagyrus* spec. nov near *sinope* and *Anagyrus mangicola* (Noyes) [15,35,36,37]. Larger hosts generally provide greater nutritional resources and improve offspring fitness, making them more suitable for female progeny production [15,18]. Accordingly, female offspring of *A. aberiae* emerged predominantly from adult female hosts, whereas smaller hosts produced mostly males. This pattern was more pronounced in the choice tests, likely because female parasitoids were able to discriminate among host stages and allocate offspring sex based on host quality and the expected fitness of the progeny. Therefore, the preference of *A. aberiae* for larger adult female hosts may have important practical implications, as the production of larger parasitoids is expected to result in higher-quality parasitoid offspring with greater potential for establishment, dispersal and population growth in the field [38,39]. Previous field studies identified peaks of *D. aberiae* female abundance in late April and again between June and July [2], suggesting that parasitoid releases during these periods could maximize parasitism rates while also promoting the production of fitter parasitoids, thereby increasing the likelihood of successful biological control. Additionally, these findings have practical implications for mass-rearing programs, as the use of adult female mealybugs is expected to improve parasitoid production efficiency.

The overall impact on pest control ultimately depends on how parasitism translates into reductions in host survival and reproductive output [16]. Parasitoids attacking late developmental stages of mealybugs have traditionally been considered less effective biological control agents, particularly among koinobiont parasitoids that do not immediately kill their hosts, thereby allowing continued host reproduction after parasitism [15,17]. Although the literature on the effects of parasitism on the reproductive potential of late-stage mealybugs is scarce, our results demonstrate that *A. aberiae* parasitism caused a rapid and substantial reduction in egg production regardless of the timing of parasitism. In many cases, oviposition ceased completely, and both host longevity and fecundity were significantly reduced. This effect was particularly pronounced in young and pre-ovipositional mature females, corresponding to the host stages preferred by *A. aberiae* in both choice and no-choice experiments. In these stages, parasitism reduced egg production by more than 93% compared with unparasitized females. A similar effect was reported for *Anagyrus pseudococci* (Girault) parasitizing pre-ovipositing *Planococcus citri* (Risso) females, where parasitized mealybugs produced only 1.4% of the eggs laid by unparasitized females [16]. The ability of *A. aberiae* to reduce egg production in females parasitized on the third day of oviposition is particularly relevant, as egg laying declined shortly after parasitism and ceased within two days, resulting in nearly 50% fewer eggs than in unparasitized females.

These findings show that *A. aberiae* strongly reduces the reproductive output of *D. aberiae*, even when parasitism occurs in ovipositing females, thereby slowing *D. aberiae* population growth despite attacking reproductively active hosts. This challenges the common assumption that parasitoids attacking late developmental stages have limited biological control potential and highlights the importance of considering reproductive suppression, in addition to host mortality, when evaluating the impact of parasitoids on pest populations.

### 4.2. Population Performance of A. aberiae

The preference of *A. aberiae* for adult female hosts was also associated with favorable demographic traits. *Anagyrus aberiae* has a shorter generation time (*T*; 24.622 vs. 67.31 days) and doubling time (*DT*; 6.98 vs. 9.19 days) than *D. aberiae*, together with higher intrinsic (*r*_m_; 0.099 vs. 0.075 day^−1^) and finite (*λ*; 1.104 vs. 1.078) rates of increase under the same experimental conditions. Taken together, these demographic parameters indicate a greater population growth potential for the parasitoid than that of its host. Among these parameters, the intrinsic rate of increase (*r*_m_) [29] is considered one of the most informative indicators of natural enemy performance, as a higher *r*_m_ than that of the target pest is generally associated with greater biological control potential [19]. Similar *r*_m_ values have been reported for other mealybug parasitoids, including *A. pseudococci*, whose *r*_m_ ranged between 0.057 and 0.147 depending on the host rearing substrate [40]. Since rearing substrate has been shown to influence parasitoid demography, differences in host rearing substrate are also likely to contribute to variation in the demographic parameters estimated for *A. aberiae*. Nevertheless, the results observed in this study support the potential of *A. aberiae* to establish and sustain populations capable of suppressing *D. aberiae* infestations. Furthermore, *A. aberiae* concentrated most of its oviposition during the first few days after emergence, and offspring produced during this period were predominantly female. This early reproductive investment suggests a strategy favoring rapid host exploitation and population increase before mortality risk increases [11]. Accordingly, providing fresh hosts to recently emerged *A. aberiae* females would maximize mass-rearing efficiency. Likewise, releasing parasitoids within the same age range is expected to enhance reproductive output and improve field performance.

### 4.3. Density-Dependent Parasitism of A. aberiae

The parasitoid’s response to different host population densities represents another key factor influencing parasitoid performance. *Anagyrus aberiae* exhibited a Type II functional response, characterized by an increase in the parasitism rate with increasing host density until saturation is reached [21]. This response type is the most common among parasitoid wasps and is generally associated with efficient exploitation of low- to intermediate-host densities [41,42]. This suggests that *A. aberiae* may contribute most effectively to pest suppression during the early stages of infestation, particularly during the first spring generations when *D. aberiae* populations remain relatively low and early suppression may prevent subsequent outbreaks. Preliminary field observations have also suggested greater parasitoid impact under these conditions (Romero et al., unpublished data). Consequently, biological control strategies should aim to establish parasitoid populations before pest densities increase, whereas severe infestations may require augmentative releases or additional pest management strategies.

Although Type III functional responses have historically been seen as more effective at stabilizing host–parasitoid dynamics because of their association with parasitoid learning and density-dependent regulation [28], recent studies suggest that behavioral adaptations, such as learning, may also occur in parasitoids exhibiting other response types [43]. Moreover, numerous parasitoids exhibiting Type II functional responses have successfully achieved partial or complete control of their host pests under field conditions, as reviewed by Fernández-Arhex and Corley [20], with *A. kamali* as an example against *Maconellicoccus hirsutus* (Green) [44].

The handling time (*T*_h_) estimated for *A. aberiae* (2.7726 h) was somewhat higher than that reported for some congeneric species. Handling times of 1.205 h and 1.8 h have been reported for *Anagyrus lopezi* (Santis) parasitizing third-instar nymphs and *Anagyrus* spec. nov near *sinope* parasitizing adult females, respectively [41,42]. In contrast, other encyrtid parasitoids such as *Aenasius bambawalei* (Hayat) have shown longer handling times, reaching 5.0012 h when parasitizing third-instar nymphs [45]. Similarly, the theoretical maximum number of hosts that could be parasitized by *A. aberiae* within a 24 h period (*T*/*T*_h_ = 8.7) was lower than that reported for *A. lopezi* and *Anagyrus* spec. nov near *sinope* with 20.2 and 13.3 adult female mealybugs, respectively, but higher than the estimated for *A. bambawalei*, with only 4.8 mealybugs [26,45]. It is important to note that functional responses are influenced by environmental conditions and experimental design, and laboratory assays should therefore be interpreted as simplified representations of more complex field interactions [20,46,47]. Handling time estimates derived from functional response models tend to overestimate the actual time devoted to parasitism, since parasitoids also spend time searching, grooming, resting, and performing other behaviors unrelated to oviposition. Therefore, incorporating direct behavioral observations may provide more realistic estimates of parasitoid foraging capacity under natural conditions [41]. Nevertheless, because the studies discussed above were also conducted under controlled laboratory conditions, the functional response parameters obtained here can be directly compared with those reported for other parasitoid species, facilitating meaningful comparisons of their relative biological control potential.

The present study identifies several key biological traits underlying the biological control potential of *A. aberiae*. Under field conditions, however, the expression of these traits will ultimately depend on their interaction with additional environmental and management factors. Climatic conditions, particularly temperature, are known to influence parasitoid performance [10,48], while pesticide applications and other agricultural practices implemented within integrated pest management programs may also affect parasitoid populations [49]. In addition, host availability and ecological interactions with other organisms may further influence biological control outcomes [50]. Future studies integrating these factors under field conditions will therefore be important to better predict the long-term performance of *A. aberiae* in commercial citrus orchards.

Overall, this study shows that the biological control potential of *A. aberiae* cannot be explained by a single trait, but rather by the combination of efficient host exploitation, rapid population growth potential and effective response to host density. Adult female mealybugs represent the most suitable host stage for parasitism, supporting the highest parasitism rates and female-biased offspring production, while also significantly reducing the pest’s reproductive output. Under field conditions, synchronizing parasitoid releases with periods of high abundance of reproductive female mealybugs may enhance pest suppression and parasitoid population establishment. In addition, the type II functional response suggests that parasitoid releases are likely to be most effective before mealybug populations reach high densities. Together, these findings provide a biological basis for optimizing the use of *A. aberiae* as a biological control agent and contribute to improving integrated pest management strategies against *D. aberiae*.

## 5. Conclusions

This study describes several ecological and demographic factors influencing the biological control potential of *A. aberiae*. The parasitoid can successfully parasitize a wide range of *D. aberiae* developmental stages, preferentially targeting adult female mealybugs, which support the highest levels of parasitism, emergence, and female offspring production. Parasitism substantially reduces host reproductive output, even when females have already initiated oviposition. In addition, *A. aberiae* exhibits favorable demographic characteristics, including an intrinsic rate of increase greater than that of its host, even when developing on late host stages such as ovipositing females, suggesting a strong capacity to establish and sustain populations under suitable conditions. When exposed to varying host densities, *A. aberiae* displayed a Type II functional response, indicating efficient exploitation of low- to intermediate-density host populations. These results demonstrate that the effectiveness of *A. aberiae* as a biological control agent relies on a combination of factors: host-stage preference, host reproductive suppression, and the capacity to maintain growing parasitoid populations. These findings improve the existing understanding of the interaction between *A. aberiae* and *D. aberiae* and provide useful information for optimizing release strategies in biological control programs and their use in integrated pest management.

## Data Availability

The data supporting the conclusions of this article will be made available by the corresponding authors upon reasonable request.
